# Testing a Model of Care for Patients on Immune Checkpoint Inhibitors Based on Electronic Patient-Reported Outcomes: Protocol for a Randomized Phase II Controlled Trial

**DOI:** 10.2196/48386

**Published:** 2023-10-18

**Authors:** André Manuel da Silva Lopes, Sara Colomer-Lahiguera, Célia Darnac, Stellio Giacomini, Sébastien Bugeia, Garance Gutknecht, Gilliosa Spurrier-Bernard, Michel Cuendet, Fanny Muet, Veronica Aedo-Lopez, Nuria Mederos, Olivier Michielin, Alfredo Addeo, Sofiya Latifyan, Manuela Eicher

**Affiliations:** 1 Institute of Higher Education and Research in Healthcare Faculty of Biology and Medicine University of Lausanne Lausanne Switzerland; 2 Department of Oncology Lausanne University Hospital Lausanne Switzerland; 3 Department of Oncology Geneva University Hospital Geneva Switzerland; 4 MelanomeFrance Teilhet France; 5 Precision Oncology Center Lausanne University Hospital Lausanne Switzerland; 6 Peter MacCallum Cancer Centre Melbourne Australia

**Keywords:** patient-reported outcomes, model of care, immune-related adverse events, remote symptom management, self-management support, self-efficacy, health-related quality of life, eHealth, telehealth, support, self-management, symptom, monitoring, cancer, electronic health record, immune, detection, questionnaire, treatment

## Abstract

**Background:**

Management of severe symptomatic immune-related adverse events (IrAEs) related to immune checkpoint inhibitors (ICIs) can be facilitated by timely detection. As patients face a heterogeneous set of symptoms outside the clinical setting, remotely monitoring and assessing symptoms by using patient-reported outcomes (PROs) may result in shorter delays between symptom onset and clinician detection.

**Objective:**

We assess the effect of a model of care for remote patient monitoring and symptom management based on PRO data on the time to detection of symptomatic IrAEs from symptom onset. The secondary objectives are to assess its effects on the time between symptomatic IrAE detection and intervention, IrAE grade (severity), health-related quality of life, self-efficacy, and overall survival at 6 months.

**Methods:**

For this study, 198 patients with cancer receiving systemic treatment comprising ICIs exclusively will be recruited from 2 Swiss university hospitals. Patients are randomized (1:1) to a digital model of care (intervention) or usual care (control group). Patients are enrolled for 6 months, and they use an electronic app to complete weekly Functional Assessment of Cancer Therapy-General questionnaire and PROMIS (PROs Measurement Information System) Self-Efficacy to Manage Symptoms questionnaires. The intervention patient group completes a standard set of 37 items in a weekly PROs version of the Common Terminology Criteria for Adverse Events (PRO-CTCAE) questionnaire, and active symptoms are reassessed daily for the first 3 months by using a modified 24-hour recall period. Patients can add items from the full PRO-CTCAE item library to their questionnaire. Nurses call patients in the event of new or worsening symptoms and manage them by using a standardized triage algorithm based on the United Kingdom Oncology Nursing Society 24-hour triage tool. This algorithm provides guidance on deciding if patients should receive in-person care, if monitoring should be increased, or if self-management education should be reinforced.

**Results:**

The Institut Suisse de Recherche Expérimentale sur le Cancer Foundation and Kaiku Health Ltd funded this study. Active recruitment began since November 2021 and is projected to conclude in November 2023. Trial results are expected to be published in the first quarter of 2024 and will be disseminated through publications submitted at international scientific conferences.

**Conclusions:**

This trial is among the first trials to use PRO data to directly influence routine care of patients treated with ICIs and addresses some limitations in previous studies. This trial collects a wider spectrum of self-reported symptom data daily. There are some methodological limitations brought by changes in evolving treatment standards for patients with cancer. This trial's results could entail further academic discussions on the challenges of diagnosing and managing symptoms associated with treatment remotely by providing further insights into the burden symptoms represent to patients and highlight the complexity of care procedures involved in managing symptomatic IrAEs.

**Trial Registration:**

ClinicalTrials.gov NCT05530187; https://www.clinicaltrials.gov/study/NCT05530187

**International Registered Report Identifier (IRRID):**

DERR1-10.2196/48386

## Introduction

Immune checkpoint inhibitors (ICIs) have increasingly become part of the standard treatment for multiple cancer types and stages, with the main benefits including superior overall survival rates [1-14]. Although ICIs are generally considered as well-tolerated, they may trigger immune-related adverse events (IrAEs), which can be severe and result in debilitating or fatal outcomes [15,16]. Treatment modality, cancer type, and patient characteristics appear to influence the likelihood of symptomatic IrAEs, which generally occur in 40%-80% of patients [10,15]. Combinations of ICI agents generally result in a higher incidence and severity of symptoms, with 55% of IrAEs being severe (grade 3 or higher) [15,17]. Even though the incidence of IrAEs appears to be positively correlated with superior objective response rates to ICIs, the occurrence of severe events may lower overall survival rates [2,18]. It is posited that solid tumors that exhibit high mutational burden elicit a stronger immune response, which may trigger more IrAEs [19,20]. Limited evidence suggests that skin, respiratory, renal, and hepatic IrAEs also appear to be correlated with melanoma, lung cancer, kidney cancer, and hepatocellular cancers, respectively, suggesting that different tumor types may increase the likelihood of specific IrAEs [21-23]. In addition, genetic risk factors such as allelic variations of *HLA-B* and mutations in the *TMEM162* gene are correlated with higher or lower likelihood of IrAEs [24]. Recent studies have highlighted that the potential chronicity of IrAEs could further burden patients and contribute to lower overall quality of life [16,25-27]. The timing of detection and intervention of IrAEs appears to play a key role in limiting their progression and outcome [10,15,28].

Although most IrAEs occur within the first 3-6 months of the start of treatment, some may develop after a year even when treatment has been discontinued [15,29,30]. In addition to the uncertainty of when they may manifest, IrAEs are heterogenous in their symptomatic presentation and are usually related to the affected tissue or organ [15]. Therefore, patients need to be ready to engage in self-care activities to self-monitor and self-manage symptoms they confront outside of the clinical environment during the multiple weeks between treatments [31].

As clinicians rely on patient recall to assess symptomatic adverse events, the time between visits and the clinician’s own judgement may inadvertently contribute to an underestimation of their severity, frequency, and burden [32,33]. Collecting patient-reported outcomes (PROs) has become a standard to accurately describe symptomatic adverse events by avoiding some of the biases of clinician reporting, thus contributing evidence on safety, tolerability, and efficacy of cancer treatments [34,35]. Recent studies show that PRO data collected electronically can enable more timely interventions to support patients in managing symptoms, resulting in improvements in overall survival, health-related quality of life (HRQoL), symptom control, and patients’ perceived self-efficacy [34,36,37]. Improvements and smaller declines in HRQoL have been observed when actionable PRO data are available to clinicians [38]. PROs can improve communication between patients and health care professionals by increasing the scope of symptoms addressed and how often they are discussed [34].

Clinical trials using electronic PROs (ePROs) in remote symptom management have noted how these data enable interventions that prevent complications related to cancer treatment, leading to similar or lower rates of hospital admissions and emergency care admissions than the current standard of care [36,37]. These models of care generally interpret ePRO symptom data that nurses use to provide personalized remote symptom management support to patients or that activate automated feedback through custom algorithms [36,37,39]. These interventions have seldom been detailed on the procedures put in place to monitor and manage ePRO symptom data. We thus hypothesized that a structured and standardized ePRO-based model of care, including remote monitoring and symptom management, using real-time data may reduce the time to detection of symptomatic IrAEs. This would, in turn, facilitate timely intervention and limit worsening of patients’ HRQoL and perceived self-efficacy to manage their conditions. This randomized controlled trial (RCT), the IePRO (IrAEs monitoring through electronic PROs) trial, aims to verify the effect of an ePRO-based model of care on the time to detection of symptomatic IrAEs.

## Methods

### Project Context

The RCT described in this protocol is the third phase of the IePRO project, which began in the year 2020. The first phase focused on the development of a PRO measure based on the PRO version of the Common Terminology Criteria for Adverse Events (PRO-CTCAE) [40]. The development of this measure has been detailed in a previously published Delphi study [41]. The second phase was concerned with the development of the ePRO-based model of care being tested in the RCT [42]. This is a 2-arm RCT taking place in the ambulatory care oncology units of 2 university hospitals in French-speaking Switzerland since 2 years. Its overall aim is to compare an ePRO-based model of care that enables remote monitoring and management of symptoms to usual care for patients with cancer receiving ICIs exclusively for up to 6 months.

### Study Population

#### Setting

Patients are being recruited in the Department of Oncology of the 2 university hospitals. They receive treatment and have same-day follow-up appointments at the hospitals’ outpatient clinics. Both sites include tumor type–oriented clinical oncology teams with treatment strategies dictated by multidisciplinary tumor boards.

#### Eligibility Criteria

Participants fulfilling all the following inclusion criteria are eligible for this study: (1) patients 18 years old or older, (2) diagnosed with cancer, (3) starting or restarting systemic single- or dual-agent ICI monotherapy in neoadjuvant, adjuvant, consolidation, or palliative settings, and (4) providing informed consent as documented by the signature on the informed consent form. The presence of any of the following exclusion criteria will lead to participant exclusion: (1) patients self-declaring not being able to use the electronic app and complete the questionnaire in French, (2) patients with any psychological, familial, or sociological condition and linguistic limitation, potentially hampering compliance with the study protocol requirements or follow-up procedures, (3) patients restarting ICI therapy who have previously participated in this study, (4) patients with cognitive impairment, as declared in the patient record, and (5) patients participating in other interventional clinical studies.

#### Screening and Enrollment

**Figure 1:**
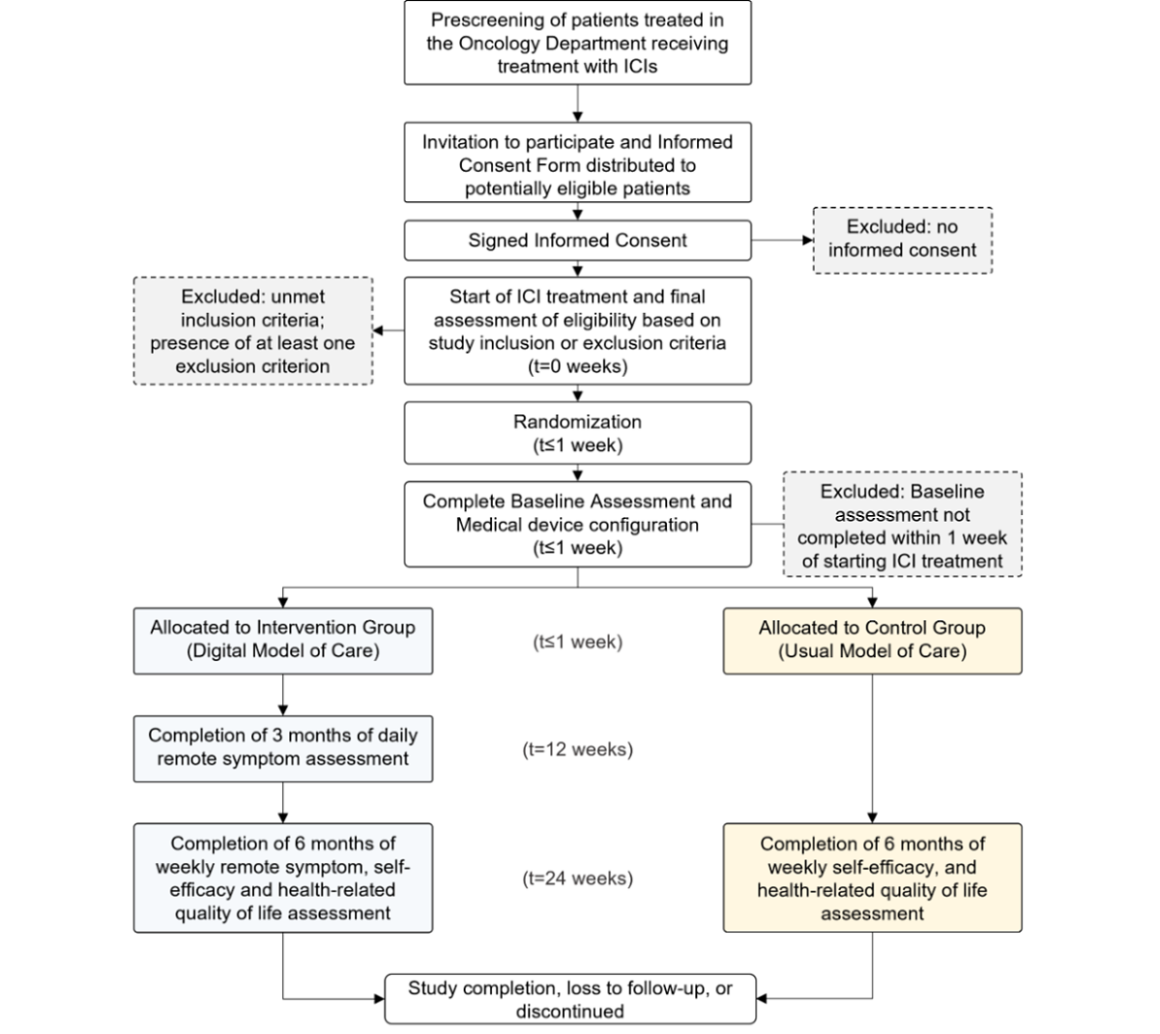
Flowchart of the enrollment of the patients in this study. ICI: immune checkpoint inhibitor.

Patients are identified by the local principal investigator and a team of sub investigators. Potentially eligible patients are approached after clinician appointments or during treatment. They are given the informed consent form (Multimedia Appendix 1), and a telephone call with a subinvestigator is scheduled within the following week to answer any questions or concerns. Patients are given at least 48 hours to consider participating in the study. Figure 1 shows the flowchart for patient enrollment in this study. Informed consent forms are collected during the patients’ next hospital appointment. Patients can be enrolled in the study up to a week after ICI treatment has started. When eligible patients refuse to participate, their reason for refusal is anonymously recorded with their consent.

### Study Design

Prior to randomization, subinvestigators collect demographic, medical history, and active treatment data from eligible consenting patients. Participants are randomized by subinvestigators using a web-based REDCap (Research Electronic Data Capture) form (version 12.4.21) [43]. The form has a randomization table (1 per site) using permuted block 1:1 randomization (block size of 4), independently prepared by the study biostatistician. Initially, the IePRO RCT was open to patients with melanoma and patients with lung cancer exclusively, and randomization was stratified by cancer type. Due to challenges in recruitment and following a protocol amendment in April 2022, this study was open to patients with all cancer types, and the stratification criteria were dropped. Blinding was not possible due to the nature of the intervention. Patients are informed that they will be randomly allocated to 1 of the 2 models of care: the standard model (control group) or the digital model (intervention group). The informed consent form and support documentation for the electronic app used to deliver the PRO questionnaires include these same conventions to further mitigate the adverse effects of the lack of blinding toward participants. Data collection instructions and reminders sent to clinicians caring for participants do not indicate which group the participant belongs to.

Patients in both groups obtain access to an electronic mobile app, which contains weekly questionnaires for HRQoL and self-efficacy to manage symptoms. An information sheet detailing how to navigate the app is also provided. Nurses and subinvestigators are available to assist patients in installing the app, registering and signing into an account, and replying to the baseline questionnaires in-person or over the phone. Patients in the intervention group have access to a symptoms questionnaire that is part of the intervention being tested. Automated reminders via emails or push notifications are sent to patients when a new questionnaire is available. Patients in both groups have the same number of scheduled clinician appointments—usually the same day and frequency of the ICI treatment—and are followed up for up to 6 months of ICI treatment.

### Intervention

**Figure 2:**
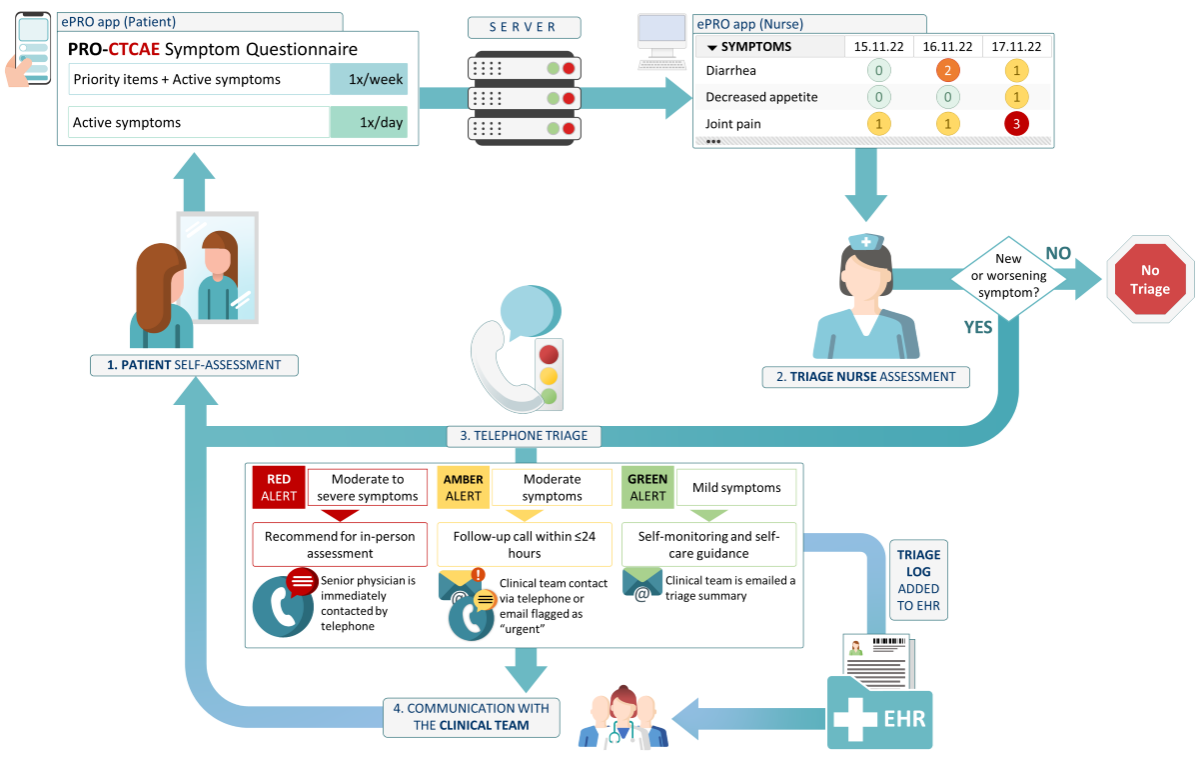
IePRO (immune-related adverse events monitoring through electronic patient-reported outcomes) Model of Care: (1) When prompted by the mobile app, patients reply to a Patient-Reported Outcomes version of the Common Terminology Criteria for Adverse Events symptom questionnaire via their own computer or mobile device. Data collected through the questionnaire are stored in a server controlled by the study sites. (2) Triage nurses are notified when patients have declared symptoms. (3) In the event of new or worsening symptoms, nurses initiate a telephone triage call with the patient. (4) Using the United Kingdom Oncology Nursing Society 24-hour triage tool, nurses determine the adequate course of action according to symptom severity and communicate the outcome of their assessments with the clinical team. The triage nurse's detailed assessment is recorded in a triage log stored in the electronic health record. EHR: electronic health record; ePRO: electronic patient-reported outcome; PRO-CTCAE: Patient-Reported Outcomes version of the Common Terminology Criteria for Adverse Events.

The intervention of this RCT consists of a complementary model of care that uses ePROs to facilitate remote symptom management [42]. This model is represented in Figure 2. Patients in the intervention arm have access to a weekly PRO-CTCAE questionnaire through an electronic mobile app. Patients receive an email invitation to access the app and an information sheet explaining its interface. Once patients access the app, they are required to complete a questionnaire of predefined symptoms. During the first 3 months of the intervention, active symptoms are reassessed daily. When replying to a questionnaire, patients can add any other item of the full PRO-CTCAE item library, which are automatically added to the following daily or weekly questionnaires as well (see Multimedia Appendix 2). The rapid and sudden onset of IrAE and ICI’s interference with pre-existing conditions and symptoms drove the decision to collect symptom data daily [10,28]. Due to the potential burden this sustained frequency could represent to patients, this was restricted to the first 3 months of the intervention. Patient replies are available in real time to the triage nurses. Two triage nurses at each site are notified via email when patients submit new replies or report severe symptoms, and they contact patients by telephone in the event of new or worsening symptoms. The nurses review patients’ answers every working day from 8 AM to noon. Outside of these hours, usual care procedures apply.

Triage nurses perform an assessment of the reported symptoms with the United Kingdom Oncology Nursing Society 24-hour triage tool [44], which are graded on a CTCAE-based scale [45]. If patients report symptoms not covered by the PRO-CTCAE questionnaire, they are also addressed during these calls. The combination of symptoms may result in different types of color-coded alerts that tie into different recommendations. Mild symptoms generally result in green alerts, where self-care and self-monitoring instructions are warranted. Moderate symptoms requiring remote follow-up within the next 24 hours correspond to an amber alert. The presence of 2 or more amber alerts or of severe symptoms results in a red alert, where urgent in-person assessment is recommended. Triage nurses communicate with physicians via email or telephone according to the type of alert. A triage log detailing the symptom assessment, recommendations to the clinical team, and the actions taken to manage patient care is added to the electronic health record (EHR).

Patients are informed that this model of care is complementary to the usual care model. They are instructed by the triage nurses and subinvestigators in the clinical team that in the event of a symptom that causes them any level of concern and would lead them to seek medical assistance, they must contact their reference clinician or the on-call oncologist as usual. It is also clarified that the likelihood of being called by the triage nurse should never delay them from seeking medical assistance. In this sense, patients are reminded of these procedures at every telephone interaction with the triage nurses.

### Electronic App (Medical Device)

This study is categorized as a clinical trial with medical device of risk category A1. It uses a web-based and mobile CE-marked app (Kaiku Health app) containing the PRO measures. The app is classed as a medical device that allows patients to submit their answers and access previous replies. Patients receive a summary of the reported symptoms detailing which of those improved, remained stable, or worsened. The app’s security features were assessed and validated by the participating hospitals’ information technology departments. Web-based and mobile versions of the app are identical in content and functionality. Patient data collected through the medical device are encrypted at rest and in transit and are subject to regular backups. Security updates are ensured by the app developer (Elekta AB). Two-factor authentication is activated for all patients.

### Study Objectives and Outcomes

#### Primary Objective and Primary Outcome

The primary objective of the IePRO RCT is to compare the effect of an ePRO-based model of care to the current standard model of care on the delay between symptomatic IrAE onset and its detection by health care providers in patients with cancer treated with ICIs. The ePRO-based model of care complements the standard model of care with remote symptom monitoring via ePROs and remote symptom management via telephone triage calls done by oncology nurses. The primary outcome is the time-to-detection of IrAEs, as evidenced by a statistically significantly lower length of time when compared to that in usual care. Time to detection is the difference expressed in days between the IrAE detection date by the clinical team and the associated symptom’s onset date according to the patient’s self-report.

#### Secondary Objectives and Secondary Outcomes

The secondary objectives of this trial include assessing the effect of the ePRO-based model of care on the following secondary outcomes: (1) the delay between symptom onset and deployment of pharmacological interventions to manage symptomatic IrAEs by a statistically significant shorter or longer average time delay, (2) the type and amount of pharmacological interventions to manage symptomatic IrAEs by a statistically significant lower average dose, (3) the average maximum symptomatic IrAE grade by a statistically lower or higher average score, (4) the HRQoL through a statistically significant higher or lower overall score of the Functional Assessment of Cancer Therapy-General (FACT-G) questionnaire [46], (5) the perceived self-efficacy to manage symptoms through a statistically significant higher or lower score of the PROMIS (PRO Measurement Information System) Self-Efficacy for Managing Chronic Conditions–Managing Symptoms (short form 8a) [47], (6) overall survival at 6 months plotted using Kaplan-Meier estimates, using routine data collected in the EHR, and (7) description of the symptomatic IrAEs experienced by patients by type and grade according to version 5.0 of the National Cancer Institute’s CTCAE [48]. Remote symptom management processes recorded by nurses will be described using (1) patient-reported symptoms triggering remote symptom management procedures using the composite grading algorithm for the PRO-CTCAE questionnaire [49] and (2) the type of issued triage alerts following triage procedures according to the United Kingdom Oncology Nursing Society 24-hour triage tool [44] and associated symptoms reported using the PRO-CTCAE questionnaire.

#### Exploratory Objectives

As exploratory objectives, the RCT will also describe the following in the intervention group: (1) symptoms reported via the PRO-CTCAE questionnaire that suggested the need for in-person intervention and patients received the intervention (true positives), (2) symptoms reported via the PRO-CTCAE questionnaire that suggested the need for in-person intervention but patients did not receive the intervention (false positives), (3) type of interventions required and provided for symptoms as reported in the triage log and the EHR, (4) symptoms reported via the PRO-CTCAE questionnaire that require an intervention beyond remote symptom management, (5) usability of the electronic app used to collect PRO symptom data and acceptability of the ePRO-based model of care, assessed through semistructured patient interviews, and (6) acceptability of the ePRO-based model of care assessed via semistructured individual interviews and focus groups with clinical nurse specialists, nurses, and physicians.

### Sample Size Calculation

A sample of 29 health records of previously treated patients with melanoma who experienced IrAEs was used to estimate the required sample size. Patients with lung cancer were not considered for this calculation due to the lack of accessibility to symptomatic IrAE data in that population. Only records where the symptom onset was reported were included for this sample size calculation. In that record sample, the time interval between symptom onset and clinician detection of symptomatic IrAEs was estimated at 4.43 days, with an SD of 3.65 days. A 2-sample *t* test with a statistical power of 90% and significance level set at .05 determined that 138 participants would be required to detect a 2-day difference, assuming the same SD of 3.65 days. Given that similar studies have reported an attrition rate of 30%, the target sample size was adjusted to 198 patients [36,50].

### Data Collection Methods

Demographic data and data relating to symptomatic IrAEs and their management for both control and intervention groups are collected through EHR review of the clinical oncologist’s follow-up notes at each scheduled appointment with participants. Data from the EHR are recorded in REDCap electronic case report forms hosted in the server of the study sponsor’s Clinical Research Center [51]. PRO data are collected through the Kaiku Health electronic app, which contains 3 questionnaires. The questionnaires were selected by the study’s subinvestigator team, including a patient advocate (GS-B), by discussing matters of burden and of pertinence of the collected data to patients, and what data were actionable for the model of care.

HRQoL data are collected via version 4 of FACT-G questionnaire, translated in French [46,52]. The questionnaire targets 4 domains: physical well-being, social or family well-being, emotional well-being, and functional well-being. Recall period, instrument scaling, scoring, and methods to handle missing data do not differ from the official administration and scoring guidelines [53]. The self-efficacy for managing symptoms is assessed weekly using the 8-item short-form French version of the PROMIS Self-Efficacy for Managing Chronic Conditions–Manage Symptoms measure [54]. Instrument scaling, scoring, and missing data handling also do not differ from the official scoring manual [55].

Symptom data in the intervention group are collected using a weekly PRO-CTCAE that includes symptom terms categorized as of the highest importance to monitor during ICI treatment. Thirty of the 37 symptom terms were identified through the Delphi study conducted in the first phase of the IePRO project [41]. The remaining 7 were selected by the IePRO project’s clinical team and principal investigators. All PRO-CTCAE items were translated in French. Currently, no official guidelines exist on how to best analyze PRO-CTCAE data longitudinally [56]. In this RCT, we will follow guidance provided by Basch et al [49] to calculate a single composite numerical grade for PRO-CTCAE symptom terms to facilitate longitudinal analysis.

As previously stated, active symptoms are reassessed daily during the first 3 months of the RCT. This is done by modifying the standard PRO-CTCAE recall period from 7 days to 24 hours. Although modifications to the recall period of PRO-CTCAE items are not encouraged, this is specifically due to potential considerable measurement errors, as that period is extended [57]. Recent studies [58,59] have highlighted how PRO measures using 24-hour recall periods more accurately convey symptom burden and variability over a short period of time, particularly for symptoms such as constipation, diarrhea, nausea, and those related to the patient’s emotional state. In patients treated with chemotherapy, shortened recall periods across different symptom PRO measures have enabled detection of clinically meaningful changes and were shown to be capable of detecting severe symptoms earlier [58,59]. Because some IrAEs can be fulminant and evolve in severity very quickly, daily reassessment of active symptoms could also support early detection and management. Although there is concern that the inclusion of all PRO-CTCAE items for daily assessment can be burdensome to patients, this concern addressed static questionnaires. In this intervention, questionnaires are dynamic and only active symptoms are reassessed daily to avoid overburdening patients.

To minimize missing data, the app requires respondents to provide a response before they are able to progress within the questionnaire. Patients receive automated reminders to reply to the questionnaires via email. In the event of early study discontinuation or withdrawal, all data collections, including PRO data, are halted. A semistructured interview guide based on the mHealth App Usability Questionnaire by Zhou et al [60] was developed to assess the usability of the ePRO app. Acceptability of the model of care will be assessed using an interview guide developed around the 7 constructs of acceptability of health care interventions as described by Sekhon et al [61]. Patient and individual staff interview data will be collected via audio recordings. Recordings will be transferred from the recording device’s secure digital card to be stored in the corresponding site’s local secured servers. Transcriptions of the recordings will be independently analyzed by 2 subinvestigators by using thematic analysis.

### Statistical Methods

In this RCT, the null hypothesis postulates that there is no difference in the primary end point (delay between symptom onset and detection be clinical team) between the intervention and control groups. The primary outcome will be analyzed comparing groups by means of multivariable regressions, adjusting for cancer type, age, and treatment regimen, against a 1-sided hypothesis. Secondary outcomes will be analyzed using multivariate regression analysis, controlled for cancer type, treatment regimen, and age. All statistical analyses will be presented as effect measure plus 95% CI, using a significance level of 5%. Variations in HRQoL and self-efficacy scores will be assessed by adjusting to baseline measure. Overall survival at 6 months will be controlled for cancer type and stage. All data processing and statistical analyses will be performed in R statistical software (v4.2.2; R Core Team 2023) and Microsoft Excel for Microsoft 365 MSO (version 2302, 2023). In-questionnaire data completeness is compulsory, though potential errors in the app may nevertheless occur. In addition, data such as time points may prove difficult to collect. According to the type and amount of missing data, strategies including imputation methods, deletions, or dismissals will be considered. For the HRQoL and self-efficacy for managing symptoms questionnaires, missing data will be handled according to official guidelines [53,55]. For exploratory outcomes, descriptive statistics will be applied. Qualitative data of semistructured interviews will be analyzed by the subinvestigators. Interviews will be transcribed verbatim and analyzed through thematic analysis.

### Patient and Public Involvement

A patient expert (GS-B) was involved from the initial stages of the design of this study. The patients helped define the outcomes of interest of the study; gave their feedback on its design, choice of instruments for PRO data collection, and choice of medical device; assisted in the creation of documents targeting patients such as the informed consent form and the information sheet for the medical device; and participated in the study’s dissemination. Ten patients gave feedback on the medical device interface during its development phase.

### Safety Reporting

The university hospitals participating in this study have signed a research agreement detailing each site’s responsibilities and data access and management procedures to ensure compliance with the research protocol and data monitoring plan. All information related to the trial will be stored securely at the corresponding study site. Documents containing participant data will be identified by a coded identification number only to maintain patient confidentiality. The sponsor and medical device developer have entered a written data processing agreement that outlines the extent in which data processing can be handled by the developer. Upon trial completion or early withdrawal, patients’ login credentials are deactivated, and PRO data are archived. The data archive is stored by the data manager and the sponsor of the study. Accessing encrypted data logs requires prior authorization from the sponsor-investigator. Trial monitoring activities are ensured by a data monitor for each site, following a clinical monitoring plan submitted alongside the research protocol to the competent ethics committee. Monitoring visits are scheduled to occur at 6, 12, 18, and 24 months (end of trial). Any study-related incidents are reported to the sponsor representative. Serious adverse events are reported to the competent ethics committee of the trial. Patients enrolled in the trial are covered by indemnity for negligent harm through the sponsor.

### Ethics Approval

This study has been approved by the ethics committee of the Canton of Vaud, Switzerland (approval 2021-00301) and is conducted according to local regulations and the Declaration of Helsinki [62]. This trial is sponsored and led by the Lausanne University Hospital in Switzerland and registered at ClinicalTrials.gov (NCT05530187). Informed consent is obtained from all participants. Due to the nature of the intervention, this study is unblinded. Data are deidentified for data analysis purposes. No compensation was provided to patients participating in this study. In addition to the major amendment to the eligibility criteria, challenges brought by the COVID-19 pandemic (including site-imposed restrictions to conducting research not related to the pandemic) and resulting uncertainty on whether all the components of this trial could be deployed and thus its original outcomes assessed, further prevented this trial from being registered prospectively. Trial registration preceded any interim data analysis, and the original outcomes have been preserved.

## Results

The first version of the research protocol of the IePRO RCT was approved in September 2021. In addition to 2 minor amendments, the latest version of the protocol contained a major amendment to the inclusion criteria, which was modified to include patients with any cancer type. This version was submitted and approved by the responsible ethics committee in April 2022. The current version of the protocol includes minor amendments that were approved in March 2023 (version 3.1 on September 22, 2022). All trial documents, including the protocol, site-specific informed consent form, and participant education materials, have been approved by the competent ethics committee. Patients have been actively recruited to this trial since November 2021, and this trial is projected to close by November 2023. The results of this trial will be published through abstracts, posters, presentations, and publications in peer-reviewed journals, when agreed to and reviewed by the principal investigators and the sponsor representative of the trial.

## Discussion

The IePRO RCT is, to our knowledge, among the first trials to use PRO data to directly influence the routine care of patients treated with ICIs. Current guidelines argue that PRO measures can guide clinicians in monitoring patients at home, optimize patient interactions when the patients come to the hospital, and highlight symptoms that could improve or resolve through supportive care interventions [32]. Crucially, PROs can help identify active treatment-associated toxicities that, if unmanaged, may worsen and require complex care, impact quality of life, lead to treatment interruptions, and thus, ultimately, decrease survival [32,34].

Recent studies have shown the feasibility and acceptability of ePRO symptom monitoring systems to monitor IrAEs but have provided limited evidence of their impact on patient care and clinical outcomes [63]. IrAE investigation and management algorithms and the lack of integration and adaptability to routine care are among the key areas requiring improvement for the success of these interventions. The triage process in the IePRO model is a potential strength, as it provides nurses with a standardized procedure to investigate and manage symptoms, potentially decreasing variations in how care and information are provided, facilitating more consistent outcomes. In addition, the model of care was conceived with adaptability in mind, to be able to accommodate 2 tertiary hospitals. The adaptive structure of the PRO-CTCAE questionnaire focuses on active symptoms and enables patients with the choice of adding self-detected symptoms, while limiting the risk of increased burden, particularly due to more frequent data collection. It is possible that its weekly data collection may more accurately portray changes throughout treatment, as opposed to multiple-month intervals of measures of HRQoL and self-efficacy for managing symptoms.

Other limitations of this study are that the broad triggers for a telephone triage call (new or worsening symptom) may lead to a significant number of calls that may not always result in meaningful changes to how care is provided. A recent publication by Msaouel et al [64] has described adaptive and more granular alert thresholds that could prevent and alleviate clinician burden. Clinicians in similar studies have noted the time-consuming nature of ePRO data review, and these broad triggers may compound that burden further and limit clinician compliance to study procedures [65]. Tolstrup et al [66] attempted to empower patients with the decision to contact the hospital, though this may have discouraged computer-naïve patients from doing so even when symptoms were concerning, leading the authors to consider a proactive approach for future iterations of remote PRO monitoring. Integration with the EHR was complicated by the differences in EHR platforms across study sites and the short time interval between the finalized code for the mobile app and the start of the study. EHR integration is a major factor for ensuring successful implementation of ePRO monitoring [63], and it remains unclear if the measures implemented to mitigate the lack thereof will be successful.

Dropping tumor type and excluding ICI agents as stratification criteria constitute a major limitation of this study, as patient groups could present important differences that could influence the type, severity, and frequency of IrAEs. This consideration should guide future analysis of the data, and its impact will be addressed in future publications. More frequent measuring and the reactive nature of the intervention increase the risk of surveillance bias, as clinicians will be more aware of the challenges patients are experiencing. The intervention also does not allow blinding of patients who may alter their usual self-monitoring and self-care behaviors due to their awareness of the triage nurse’s monitoring [66]. As this study is being deployed in 2 sites, tracking hospitalization and emergency room admissions for patients in the control group was outside the resources available to the study team. As a consequence, they were not included as secondary end points for this study, limiting insight on its efficiency to improved care. Overall survival measures were capped at 6 months due to limited resources to pursue a longer target, which could hinder the visibility of long-term effects of the model of care. Lastly, excluding patients who self-declare unable to use the electronic app and the lack of alternative means to self-report symptoms limit the comparability of this sample to the general population [36,67]. Despite these limitations, the IePRO RCT takes a pragmatic approach to symptom monitoring with ePRO data by not insulating its model of care from the existing resources the sites use to assist patients. Importantly, the IePRO RCT may further highlight the challenges patients treated with ICIs face outside the clinical setting, particularly as more data revealing the true burden of treatment-associated toxicities continue to emerge.

## Appendix

Multimedia Appendix 1 Informed consent form (in French).

Multimedia Appendix 2 Symptom Questionnaire flowchart demonstrating how the electronic app uses symptoms that the patient declares as active to then requesting a reassessment in the following day by using a modified 24-hour recall period. It also shows how symptoms that emerge between weekly questionnaires are taken into account in the next weekly questionnaire even when the symptoms are no longer active.

## Abbreviations

CTCAE: Common Terminology Criteria for Adverse Events
EHR: electronic health record
ePRO: electronic patient-reported outcome
FACT-G: Functional Assessment of Cancer Therapy-General
HRQoL: health-related quality of life
IePRO: immune-related adverse events monitoring through electronic patient-reported outcomes
ICI: immune checkpoint inhibitor
IrAE: immune-related adverse event
PRO: patient-reported outcome
PRO-CTCAE: Patient-Reported Outcomes version of the Common Terminology Criteria for Adverse Events
PROMIS: Patient-Reported Outcomes Measurement Information System
RCT: randomized controlled trial
REDCap: Research Electronic Data Capture

## Data Availability

The data sets generated during or analyzed during this study are not publicly available due to its sensitive nature but are available from the corresponding author on reasonable request.
