# Supplementary material for: Testing a Model of Care for Patients on Immune Checkpoint Inhibitors Based on Electronic Patient-Reported Outcomes: Protocol for a Randomized Phase II Controlled Trial
Source: JMIR Res Protoc. 2023 Oct 18;12:e48386. doi: 10.2196/48386 (PMC10620631; doi:10.2196/48386)
Supplement: Multimedia Appendix 1 [file resprot_v12i1e48386_app1.pdf]

Multimedia Appendix 1 – Model informed consent form (in French)

Test d'un modèle de soins basé sur les symptômes rapportés électroniquement par les patients atteints d'un cancer et traités avec des inhibiteurs du point de contrôle immunitaire: une étude de phase II = IePRO

Cette étude est organisée par : Prof. Manuela Eicher, directrice UNIL-CHUV

Madame, Monsieur,

Nous vous proposons de participer à notre projet de recherche. Cette feuille d'information décrit le projet de recherche, d'abord dans une version courte (résumé), comme s'il s'agissait d'une table des matières, puis dans une version longue (version détaillée).

Résumé

1	**Objectifs de l'étude**<br>Par la présente, nous vous proposons de participer à notre étude clinique portant sur un modèle de prise en charge complémentaire aux soins standards. Cette étude concerne les patient atteints d'un cancer, traités avec une immunothérapie par inhibiteurs du point de contrôle immunitaire. Nous effectuons cette étude pour vérifier l'efficacité d'un nouveau modèle de soins pour la détection précoce des effets indésirables liés au traitement. Ce modèle de soins utilise les symptômes rapportés par les patients à l'aide d'une application électronique. L'objectif de cette étude est de déterminer si ce modèle pourrait permettre une intervention plus rapide et une meilleure gestion des éventuelles complications liées au traitement.
2	**Sélection des personnes**<br>Vous souffrez d'un cancer et vous êtes sous un traitement d'immunothérapie par des inhibiteurs du point de contrôle immunitaire. C'est la raison pour laquelle nous vous faisons parvenir cette feuille d'information.
3	**Informations générales sur le projet**<br>Cette étude se base sur l'utilisation d'une application électronique (disponible sur l'ordinateur, tablette ou smartphone) qui vous permettra de signaler vos symptômes, d'évaluer votre sentiment d'auto-efficacité pour les gérer et la perception de votre qualité de vie, au moyen d'un questionnaire, à distance. Cette application a un marquage CE (conformité européenne).<br><br>Si vous décidez de participer à cette étude vous serez répartis aléatoirement sur deux groupes, correspondant à deux méthodes de gestion des symptômes : le groupe « soins standards » et le groupe « numérique ». Cette répartition est faite automatiquement sur un ordinateur, une fois que vous aurez donné votre consentement pour l'étude. Vous aurez la même probabilité d'être répartis dans l'un des deux groupes.<br><br>La durée de participation est de 6 mois et l'étude sera réalisée sur deux sites, dans les départements d'oncologie du Centre Hospitalier Universitaire Vaudois (CHUV) et des Hôpitaux Universitaires de Genève (HUG). Sur l'ensemble des sites, 198 patients participeront à cette étude.
4	**Déroulement pour les participants**<br>Tous les participants de l'étude auront le même nombre de consultations et le même accès aux soins que les personnes qui ne participent pas à cette étude. Les modalités de prise en charge dans cette étude sont complémentaires à la prise en charge habituelle.<br><br>Les deux groupes de participants auront accès à une application électronique avec deux questionnaires : un sur leur sentiment d'auto-efficacité (« auto-confiance ») à gérer les

	symptômes et un sur la qualité de vie. Ces questionnaires sont à remplir une fois par semaine et prennent environ 15 minutes.<br><br>Au-delà des soins normaux, si vous êtes attribués au groupe « soins standards », vous répondrez à ces deux questionnaires une fois par semaine pendant six mois.<br><br>**Si vous êtes attribué au groupe « numérique »,** vous aurez accès aux mêmes questionnaires que le groupe « soins standards » et à un questionnaire supplémentaire qui vous permettra de déclarer vos symptômes liés au traitement :<br>• Pendant les 3 premiers mois de l'étude, l'application vous demandera de réévaluer vos symptômes actifs tous les jours, avec une version courte du questionnaire (« questionnaire journalier »).<br>• À partir du quatrième mois et jusqu'à la fin de l'étude, vous répondrez uniquement à une version hebdomadaire, une fois par semaine.<br>Une équipe d'infirmier·ère·s relèvera les symptômes déclarés et contactera les participants par téléphone pour les soutenir dans la gestion des symptômes, les jours ouvrables entre 8h et 12h. En dehors de ces horaires, vous devrez contacter le médecin oncologue de garde. En aucun cas ces mesures doivent remplacer un appel à votre médecin oncologue, et vous devrez le contacter directement si vous êtes concerné par un ou plusieurs symptômes, ou si vous avez des questions concernant vos symptômes et votre traitement.
5	**Bénéfices pour les participants**<br>Si vous participez à l'étude, cela pourra éventuellement vous aider à développer des connaissances plus approfondies sur les méthodes de surveillance et de gestion des effets indésirables liés au traitement avec une immunothérapie par inhibiteurs du point de contrôle immunitaire. Et il se peut que vous tiriez un bénéfice dans la prise en charge de vos symptômes. Les résultats de l'étude pourraient se révéler importants par la suite pour les personnes qui seront touchées par la même maladie que vous.
6	**Droits des participants**<br>Vous êtes libre d'accepter ou de refuser de participer à l'étude. Si vous décidez de ne pas participer, cela ne changera rien à votre prise en charge médicale. Vous n'avez pas à justifier vos décisions.
7	**Obligations des participants**<br>Si vous décidez de participer à l'étude, vous devrez observer certaines règles :<br>▪ Vous devrez informer votre médecin et/ou équipe clinique de tout nouveau symptôme ou nouveau trouble, et de tout changement dans votre état de santé. **L'information que vous rapportez dans cette étude NE SE SUBSTITUE PAS à la communication avec votre médecin / équipe clinique**.<br>▪ Vous devrez poursuivre les instructions médicales de votre oncologue et de l'équipe clinique.<br>▪ Vous devrez suivre le plan de l'étude (remplissage des questionnaires, consultations téléphoniques et présentielles, entretiens, aux dates indiquées) pour son bon déroulement.
8	**Risques**<br>La participation à cette étude ne comporte pas de risques si ce n'est éventuellement des risques mineurs liés à la charge émotionnelle de participer aux entretiens et de compléter les questionnaires. Il se peut que certaines questions, présentes dans les questionnaires ou discutées lors des entretiens, puissent vous affecter émotionnellement. Vous pourriez, en effet, prendre conscience d'éventuelles difficultés ressenties et éprouver une certaine charge émotionnelle à cet égard. Vous pourriez en autre avoir un faux sentiment de sécurité en utilisant l'application ePRO en vous attendant à recevoir une réponse immédiate de l'équipe d'oncologie en cas de survenue de symptômes graves. En cas de symptômes perçus comme graves, veuillez contacter directement l'équipe de oncologie. D'autres risques encore inconnus peuvent également exister.

9	**Confidentialité des données et des échantillons**<br>Nous respectons toutes les dispositions légales relatives à la protection des données. Toutes les personnes impliquées sont soumises au secret professionnel. Vos données personnelles et médicales seront protégées et utilisées sous une forme codée. Les données vous concernant pourront être réutilisées dans d’autres projets de recherche si vous y consentez expressément en signant le document prévu à cet effet.
10	**Retrait de l’étude**<br>Vous pouvez à tout moment vous retirer du projet si vous le souhaitez. Les données médicales recueillies jusque-là seront analysées malgré tout.
11	**Compensation des participants**<br>Si vous participez à cette étude, vous ne recevrez pour cela aucune compensation.
12	**Réparation des dommages subis**<br>Le Centre Hospitalier Universitaire Vaudois (CHUV) (promoteur) qui a initié l'étude et est en charge de sa réalisation, est responsable des dommages que vous pourriez subir en relation avec les activités de recherche.
13	**Financement de l’étude**<br>L’étude est financée par l’Institut Suisse de Recherche Expérimentale sur le Cancer (ISREC) et par Kaiku Health Ltd.
14	**Interlocuteur(s)**<br>Vous pouvez à tout moment poser toutes vos questions et demander toutes les précisions nécessaires aux personnes suivantes :<br><br>**André Lopes (collaborateur scientifique)**<br>[Contact information redacted for publication]<br><br>**Stellio Giacomini (collaborateur scientifique)**<br>[Contact information redacted for publication]<br><br>**Célia Darnac (collaboratrice scientifique)**<br>[Contact information redacted for publication]<br><br>**Dr. Sofiya Latifyan (Investigatrice Principale):**<br>[Contact information redacted for publication]<br><br>**Prof. Manuela Eicher (co-Investigatrice et représentante du Sponsor)**<br>[Contact information redacted for publication]

Information détaillée

1. Objectifs de l’étude

Nous vous remercions de votre intérêt et de votre participation à cette étude. Cette étude devrait nous permettre de savoir dans quelle mesure une surveillance à distance complémentaire des symptômes est utile dans la détection précoce des effets indésirables liés du traitement, améliorant la prise en charge des patients traités avec des inhibiteurs de points de contrôle immunitaire.

2. Sélection des personnes pouvant participer à l’étude

La participation est ouverte à toutes les personnes de 18 ans ou plus, et souffrant d’un cancer traité avec des inhibiteurs du point de contrôle immunitaire au département d’oncologie du Centre Hospitalier Universitaire Vaudois (DO-CHUV) ou des Hôpitaux Universitaires de Genève (HUG). Elle est en revanche fermée aux personnes qui ne se considèrent pas capables d’utiliser un outil électronique (sur un smartphone, tablette ou ordinateur) avec des questionnaires en langue française. Sont également exclues de l’étude les personnes diagnostiquées avec des

perturbations cognitives, ainsi que des limitations psychologiques, sociologiques ou linguistiques qui pourraient empêcher les personnes de répondre aux obligations de l'étude. Les personnes inscrites dans d'autres études cliniques interventionnelles sont aussi exclues.

3. Informations générales sur l'étude

- Vous allez recevoir un traitement d'immunothérapie par des inhibiteurs de point de contrôle immunitaire. Ce traitement peut produire des effets indésirables, manifestés par certains symptômes, qui doivent être surveillés et traités.
- Cette étude propose une prise en charge complémentaire aux soins normaux, pour vérifier si les effets indésirables du traitement peuvent être détectés plus rapidement et surveillés d'une manière plus efficace.
- L'étude se base sur l'utilisation d'une application électronique (disponible sur l'ordinateur, tablette ou smartphone) qui vous permettra de signaler vos symptômes, d'évaluer votre sentiment d'auto-efficacité à les gérer et la perception de votre qualité de vie, au travers d'un questionnaire, à distance. Cette application a un Marquage CE (conformité européenne).
- Si vous décidez de participer à cette étude, vous serez répartis aléatoirement dans un des deux groupes, correspondant à deux méthodes distinctes de gestion des symptômes : le groupe « soins standards » et le groupe « numérique ». Cette répartition sera faite automatiquement par un ordinateur, lorsque que vous aurez donné votre consentement pour l'étude. Vous aurez la même probabilité d'être répartis dans l'un des deux groupes.
- La durée de participation est de 6 mois, afin de collecter suffisamment de données pour répondre aux objectifs de l'étude. Une fois terminée, votre prise en charge se maintiendra selon les procédures standards. Cette étude sera réalisée sur deux sites, dans les départements d'oncologie du Centre Hospitalier Universitaire Vaudois (CHUV) et des Hôpitaux Universitaires de Genève (HUG). Sur l'ensemble des sites, 198 patients participeront à cette étude.

- Nous effectuons cette étude dans le respect des prescriptions de la législation Suisse. Nous suivons en outre l'ensemble des directives reconnues au niveau international et de la commission cantonale d'éthique compétente.
- Vous trouverez aussi un descriptif de l'étude sur le site Internet de l'Office Fédéral de la Santé Publique : www.kofam.ch

4. Déroulement pour les participants

Si vous acceptez de participer à l'étude, vous serez aléatoirement attribué à l'un des deux groupes de l'étude (groupe « soins standards » et groupe « numérique »). Vous trouverez à la fin de cette feuille d'information un schéma du déroulement de l'étude.

<u>Pour les participants du groupe « soins standards » :</u>

Vous serez informé par votre oncologue des potentiels effets secondaires du traitement et recevrez des consignes pour la gestion des symptômes. Ensuite, l'investigateur vous donnera accès à deux questionnaires électroniques auxquels vous pourrez accéder à distance, en utilisant votre smartphone, tablette ou un ordinateur connecté à internet. L'investigateur vous aidera à configurer et utiliser ces questionnaires. Les questionnaires sont :

- Questionnaire sur la qualité de vie : composé de 27 questions, qui devrait vous prendre environ 10 minutes à remplir.
- Questionnaire sur l'auto-efficacité à gérer les symptômes : composé de 8 questions, qui devrait vous prendre environ 5 minutes à remplir.

Ces questionnaires seront à remplir une fois par semaine à intervalles réguliers, à distance. À tout moment, si vous présentez des symptômes qui vous inquiètent, vous devez contacter l'oncologue de garde par téléphone.

<u>Pour les participants du groupe « numérique » :</u>

Comme pour les participants du groupe « soins standard », vous serez informé par votre oncologue des potentiels effets secondaires du traitement, et vous recevrez des consignes pour la gestion des symptômes. Ensuite, l'investigateur vous donnera accès à trois questionnaires électroniques auxquels vous pourrez accéder à distance en utilisant votre smartphone, tablette ou un ordinateur connecté à internet. Les questionnaires sont :

- Questionnaire sur **la qualité de vie** : composé de 27 questions, qui devrait vous prendre environ 10 minutes à remplir, une fois par semaine, à intervalles réguliers.
- Questionnaire sur **l'auto-efficacité pour gérer des symptômes** : composé de 8 questions, qui devrait vous prendre environ 5 minutes à remplir, une fois par semaine, à intervalles réguliers.
- Questionnaire sur **les symptômes** : ce questionnaire a deux versions – une hebdomadaire et une journalière :
  - Version hebdomadaire : composée de 70 questions, qui devrait vous prendre environ 20 minutes à remplir, une fois par semaine, à intervalles réguliers.
  - Version journalière : composée uniquement des symptômes que vous avez signalés dans le questionnaire précédent (nombre de questions variable), à remplir tous les jours. Si vous n'avez pas déclaré des symptômes avant, l'application vous permettra d'en ajouter tous les jours. Cette version est à remplir que pendant les trois premiers mois de l'étude.

Une fois le questionnaire sur les symptômes remplit, une équipe d'infirmier·ère·s du département d'oncologie relèvera les symptômes déclarés. Selon les symptômes déclarés, l'équipe peut vous contacter par téléphone pour vous soutenir dans la gestion des symptômes. Ces appels sont faits uniquement pendant les jours ouvrables, de 8h à 12h. Leurs indications peuvent inclure des conseils pour minimiser les symptômes et aller jusqu'à une demande de vous présenter à l'hôpital pour une évaluation présentielle.

En dehors des horaires mentionnés ci-dessus, vous devez contacter l'oncologue de garde en cas de questions concernant vos symptômes.

En aucun cas ces mesures remplacent un appel à votre médecin oncologue et vous devrez le contacter directement si vous êtes concerné par un ou plusieurs symptômes, ou si vous avez des questions sur vos symptômes et sur votre traitement.

Lors de la visite de fin d'étude nous vous demanderons de participer à un entretien d'environ 1 heure afin d'évaluer l'ergonomie de l'application ePRO et votre expérience de la prise en charge.

Une fois l'étude terminée, votre prise en charge sera assurée par le médecin oncologue. À tout moment de cette étude et après qu'elle soit terminée, si vous présentez des symptômes qui vous inquiètent, vous devrez contacter votre oncologue aux horaires de bureau ou l'oncologue de garde par téléphone en dehors des horaires de bureau.

Il se peut que nous devions vous retirer de l'étude avant le terme prévu. Cette situation peut se produire si votre médecin change de traitement anti-cancéreux ou si vous êtes hospitalisé pour une longue période pendant la durée de l'étude.

En pareil cas, après désactivation de votre compte utilisateur, nous vous proposerons de désinstaller l'application électronique de vos appareils personnels.

5. Bénéfices pour les participants

Si vous participez à l'étude, cela pourra éventuellement vous aider à développer des connaissances plus approfondies sur les méthodes de surveillance et gestion des effets indésirables liées au traitement avec une immunothérapie par inhibiteurs du point de contrôle immunitaire. Et il se peut que vous tiriez un bénéfice dans la prise en charge de vos symptômes. Les résultats de l'étude pourraient se révéler importants par la suite pour les personnes touchées par la même maladie que vous. Nous vous remercions de votre intérêt et de votre participation à cette étude.

6. Droits des participants

Votre participation est entièrement libre. Si vous choisissez de ne pas participer ou si vous choisissez de participer et revenez sur votre décision pendant le déroulement de l'étude, vous n'aurez pas à justifier votre refus. Cela ne changera rien à votre prise en charge médicale habituelle. Vous pouvez à tout moment poser toutes les questions nécessaires au sujet de l'étude. Veuillez-vous adresser pour ce faire à la personne indiquée à la fin de la présente feuille d'information.

7. Obligations des participants

Pour répondre à des critères standards de qualité de l'étude, chaque participant doit correspondre à certaines obligations. En tant que participant à l'étude, vous serez tenu :

- de suivre les instructions médicales de votre oncologue et de vous conformer au plan de l'étude;
- de suivre les indications des infirmier·ère·s si vous intégrez le groupe « numérique » ;
- d'informer votre personne de contact pour l'étude de l'évolution de la maladie et de lui signaler tout nouveau symptôme, tout nouveau trouble et tout changement dans votre état ;
- d'informer votre personne de contact pour l'étude de tout traitement ou thérapie concomitant·e, prescrit·e par un autre médecin ; de l'informer également de tous les médicaments que vous prenez ;
- d'informer votre personne de contact pour l'étude si vous changez l'appareil électronique que vous utilisez habituellement pour remplir le questionnaire (smartphone, tablette, ordinateur).

8. Risques et contraintes pour les participants

La participation à cette étude ne comporte pas de risques, si ce n'est éventuellement des risques mineurs liés à la charge émotionnelle de participer aux entretiens et de compléter les questionnaires. Il se peut que certaines questions, présentes dans les questionnaires ou discutées lors des entretiens, puissent vous affecter émotionnellement. Vous pourriez, en effet, prendre conscience d'éventuelles difficultés ressenties et éprouver une certaine charge émotionnelle à cet égard.
Vous pourriez en autre avoir un faux sentiment de sécurité en utilisant l'application ePRO en vous attendant à recevoir une réponse immédiate de votre infirmière investigatrice en cas de survenue de symptômes graves. En cas de symptômes perçus comme graves, veuillez contacter directement votre médecin oncologue.
D'autres risques encore inconnus peuvent également exister. Si vous avez besoin d'exprimer ces émotions, et que vous n'êtes pas à l'aise pour les partager avec l'équipe de l'étude, vous pouvez prendre contact avec la psycho-oncologue du département d'oncologie :

[Contact information redacted for publication]

9. Découvertes pendant l'étude

L'investigateur vous avisera pendant l'étude de toute nouvelle découverte susceptible d'influer sur les bénéfices de l'étude ou votre sécurité, et donc sur votre consentement à participer. Vous serez informé oralement et par écrit.

10. Confidentialité des données et des échantillons

Pour les besoins de l'étude, nous enregistrerons vos données personnelles et médicales. Seul un nombre limité de personnes pourront consulter vos données sous une forme non codée, et exclusivement afin de pouvoir accomplir des tâches nécessaires au déroulement du projet. Les données recueillies à des fins de recherche sont codées lors de leur collecte. Le codage signifie que toutes les données permettant de vous identifier (p. ex. le nom, la date de naissance, etc.) sont remplacées par un code. Le code reste en permanence au sein de l'institution / de l'hôpital. Les personnes ne connaissant pas ce code ne peuvent pas lier ces données à votre personne. Dans le cas d'une publication, les données agrégées ne vous sont donc pas imputables en tant que personne. Votre nom n'apparaîtra jamais sur Internet ou dans une publication. Parfois, les

journaux scientifiques exigent la transmission de données individuelles (données brutes). Si des données individuelles doivent être transmises, elles sont toujours codées et ne permettront donc pas de vous identifier en tant que personne. Toutes les personnes impliquées dans l'étude de quelque manière que ce soit sont tenues au secret professionnel. Toutes les directives relatives à la protection des données seront respectées et vous aurez à tout moment le droit de consulter vos données.

Durant son déroulement, l'étude peut faire l'objet d'inspections. Celles-ci peuvent être effectuées par la commission d'éthique qui s'est chargée de son contrôle initial et l'a autorisé, par l'autorité suisse de contrôle et d'autorisation des produits thérapeutiques Swissmedic ou par l'organisme qui l'a initiée. Il se peut que l'investigateur doive communiquer vos données personnelles et médicales pour les besoins de ces inspections.
Il est possible que le médecin s'occupant de votre suivi médical soit contacté au sujet de votre état de santé.

Pour les patients participants du groupe « numérique », les données personnelles utilisées par l'application électronique seront encryptées et stockées sur un serveur en Allemagne géré par Google, Inc, sous la plateforme Google Cloud. Ces données pourront uniquement être décryptées avec l'autorisation de la personne responsable de l'étude IePRO, Prof. Manuela Eicher. À la fin de l'étude, ces données seront supprimées du serveur.

11. Retrait de d'étude

Vous pouvez à tout moment vous retirer de l'étude si vous le souhaitez. Les données médicales recueillis jusque-là seront tout de même analysées, ceci afin de ne pas compromettre la valeur de l'étude dans son ensemble.
Il est impossible de rendre vos données anonymes, c'est pour cela qu'elles resteront codées. Vous devez donc être d'accord avec cela avant de donner votre consentement.

12. Compensation des participants

Si vous participez à cette étude, vous ne recevrez pour cela aucune compensation financière.
Votre participation n'aura aucune conséquence financière pour vous ou votre assurance maladie.

13. Réparation des dommages subis

Le Centre Hospitalier Universitaire Vaudois (CHUV) (promoteur) qui a initié l'étude et est en charge de sa réalisation, est responsable des dommages que vous pourriez subir en relation avec les activités de recherche. Les conditions et la procédure sont fixées par la loi.
Pour les dommages occasionnés par un dispositif médical approuvé et employé selon les standards médicaux ou qui seraient également survenus lors d'un traitement avec une thérapie conventionnelle, les règles de responsabilité applicables sont celles régissant les traitements en dehors d'une étude.
Si vous avez subi un dommage, veuillez-vous adresser à l'investigateur responsable du projet.

14. Financement de l'étude

L'étude est financée par l'Institut Suisse de Recherche Expérimentale sur le Cancer (ISREC) et par Kaiku Health Ltd.

15. Interlocuteur(s)

En cas de doute, de craintes ou d'urgences pendant ou après l'étude, vous pouvez vous adresser à tout moment à l'un des interlocuteurs suivants :

André Lopes (collaborateur scientifique)
[Contact information redacted for publication]

Stellio Giacomini (collaborateur scientifique)
[Contact information redacted for publication]

Célia Darnac (collaboratrice scientifique)
[Contact information redacted for publication]

Dr. Sofiya Latifyan (Investigatrice Principale):
[Contact information redacted for publication]

Prof. Manuela Eicher (co-Investigatrice et représentante du Sponsor)
[Contact information redacted for publication]

16. Glossaire (termes nécessitant une explication)

- **Qu'entend-on par « auto-efficacité » ?**

L'auto-efficacité correspond aux croyances d'un individu par rapport à sa capacité de réaliser une tâche, un apprentissage, un défi ou un changement avec succès. Dans le contexte spécifique de cette étude, il s'agit des croyances des participants par rapport à leur capacité de gérer leurs symptômes.

- **Qu'entend-on par « immunothérapie par inhibiteurs du point de contrôle immunitaire » ?**

L'immunothérapie par inhibiteurs du point de contrôle immunitaire est un type de traitement anti-cancéreux qui augmente la réponse du système immunitaire au cancer. Ce type de traitement est de plus en plus utilisé contre différents types de cancer.
Le rôle des points de contrôle du système immunitaire est de limiter la réponse du système immunitaire afin de ne pas endommager les cellules saines. Malheureusement, les cellules cancéreuses exploitent ce mécanisme au point de désactiver le système immunitaire, permettant au cancer de progresser. Les inhibiteurs du point de contrôle immunitaire évitent que le cancer puisse profiter de ce mécanisme.

Déroulement de l'étude

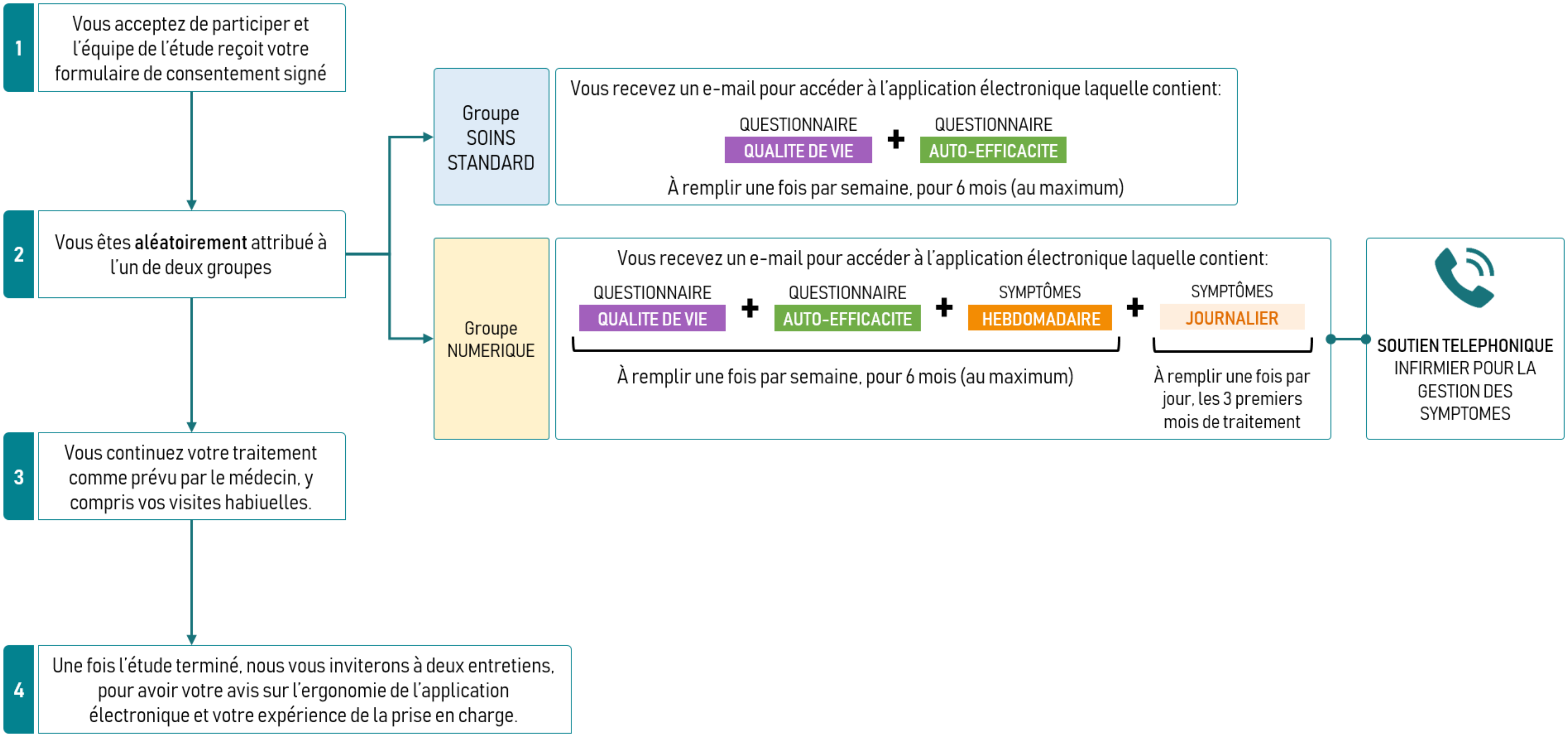

Déclaration de consentement

Déclaration de consentement écrite pour la participation à un projet de recherche

Veuillez lire attentivement ce formulaire. N'hésitez pas à poser des questions lorsque vous ne comprenez pas quelque chose ou que vous souhaitez avoir des précisions.

**NUMÉRO BASEC DE L'ÉTUDE: (APRÈS SOUMISSION À LA COMMISSION D'ÉTHIQUE COMPÉTENTE) :**	2021-00301
**TITRE DE L'ÉTUDE : (TITRE SCIENTIFIQUE ET TITRE USUEL)**	Test d'un modèle de soins basé sur les symptômes rapportés électroniquement par les patients atteints d'un cancer et traités avec des inhibiteurs du point de contrôle immunitaire: une étude de phase II (IePRO)
**Institution responsable :** (Promoteur avec adresse complète) :	Centre Hospitalier Universitaire Vaudois, Rue du Bugnon 46, 1011 Lausanne
**LIEU DE RÉALISATION DE L'ÉTUDE:**	
**Médecin responsable du projet sur le site :** (nom et prénom en caractères d'imprimerie) :	
**Participant / participante :** (nom et prénom en caractères d'imprimerie) : Date de naissance :	

- Je déclare avoir été informé, par l'investigateur responsable de cette étude soussigné, oralement et par écrit, des objectifs et du déroulement de l'étude ainsi que des effets présumés, des avantages, des inconvénients possibles et des risques éventuels.
- Je prends part à cette étude de façon volontaire et j'accepte le contenu de la feuille d'information qui m'a été remise sur l'étude précitée. J'ai eu suffisamment de temps pour prendre ma décision.
- J'ai reçu des réponses satisfaisantes aux questions que j'ai posées en relation avec ma participation à l'étude. Je conserve la feuille d'information et reçois une copie de ma déclaration de consentement écrite.
- J'accepte que les spécialistes compétents du promoteur de l'étude, de la Commission d'éthique compétente et de l'autorité suisse de contrôle et d'autorisation des produits thérapeutiques Swissmedic, puissent consulter mes données brutes afin de procéder à des contrôles, à condition toutefois que la confidentialité de ces données soit strictement assurée.
- Je sais que mes données personnelles peuvent être transmises / transmis à des fins de recherche dans le cadre de ce projet uniquement et sous une forme codée, aussi à l'étranger.
- Je peux, à tout moment et sans avoir à me justifier, révoquer mon consentement à participer à l'étude, sans que cela n'ait de répercussion défavorable sur la suite de ma prise en charge. Les données médicales qui ont été recueillis jusque-là seront cependant analysés.
- Je suis informé que le promoteur couvre les dommages éventuels que je pourrais subir imputables au projet.
- Je suis conscient que les obligations mentionnées dans la feuille d'information destinée aux participants doivent être respectées pendant toute la durée de l'étude. La direction de l'étude peut m'en exclure à tout moment dans l'intérêt de ma santé.

Lieu, date	Signature du participant / de la participante

Attestation de l’investigateur : Par la présente, j’atteste avoir expliqué au participant / à la participante la nature, l’importance et la portée de l’étude. Je déclare satisfaire à toutes les obligations en relation avec ce projet conformément au droit en vigueur. Si je devais prendre connaissance, à quelque moment que ce soit durant la réalisation du projet, d’éléments susceptibles d’influer sur le consentement du participant / de la participante à prendre part au projet, je m’engage à l’en informer immédiatement.

Lieu, date	Nom et prénom de l’investigateur assurant l’information aux participants en caractères d’imprimerie.<br><br>Signature de l’investigateur
