# Supplementary figures and images for: Testing a Model of Care for Patients on Immune Checkpoint Inhibitors Based on Electronic Patient-Reported Outcomes: Protocol for a Randomized Phase II Controlled Trial
Source: JMIR Res Protoc. 2023 Oct 18;12:e48386. doi: 10.2196/48386 (PMC10620631; doi:10.2196/48386)

Multimedia Appendix 2 – Symptom questionnaire flow

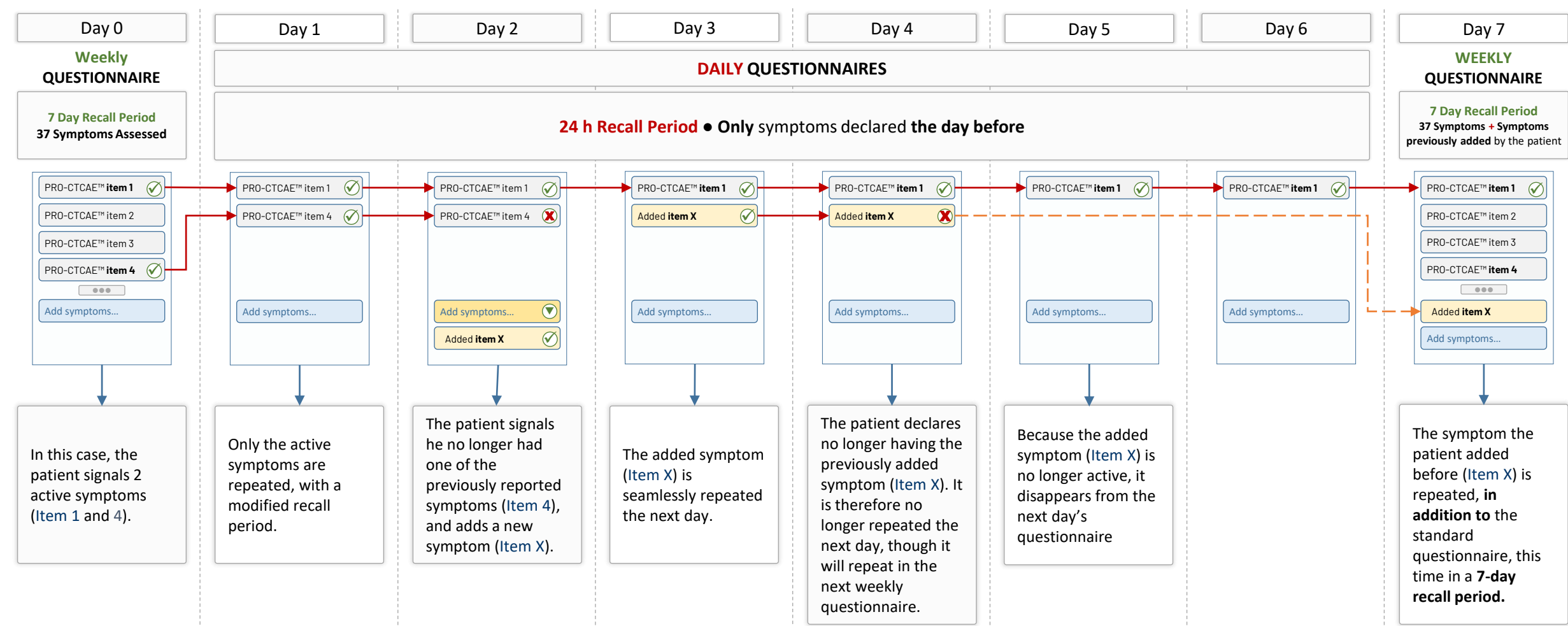

Supplement: Multimedia Appendix 2 [file resprot_v12i1e48386_app2.pdf]
